# Time to move beyond monological perspectives in health behavior change communication research and practice

**DOI:** 10.3389/fpsyg.2023.1070006

**Published:** 2023-06-05

**Authors:** Antje Maria Schöps, Timothy Charles Skinner, Christina Fogtmann Fosgerau

**Affiliations:** ^1^Department of Nordic Studies and Linguistics, University of Copenhagen, Copenhagen, Denmark; ^2^Department of Psychology, University of Copenhagen, Copenhagen, Denmark; ^3^Department of Psychology, La Trobe University, Bendigo, VIC, Australia; ^4^Centre for Behavioural Research in Diabetes, Melbourne, VIC, Australia

**Keywords:** health behavior, behavior change, communication, health care, language

## Abstract

Chronic disease self-management and health behavior change programs are becoming increasingly important to health service delivery to prevent the development of chronic disease and optimize health outcomes for those who suffer from it. To train people to deliver these programs effectively, we need to understand both the what and how of program delivery. While there is an abundant literature on what, and a merging evidence about what techniques to use, such as goals setting and self-monitoring, the literature on how programs should be delivered is less well developed. This paper reviews emerging research in this area and identifies an underlying monological approach. We argue that this currently dominant model cannot address the key issues in this area. By considering the theoretical framework of Dialogism, we introduce the method of Conversation Analysis to the field of behavior change interventions. Extensive research into health communication has endeavored to show the importance of language and the organization of interactions. We demonstrate and discuss how a monological approach to interventions prevents exploration of what professionals do to deliver intervention content. In doing so, we show that techniques do not account for how successfully an intervention is delivered.

## Introduction

Chronic diseases such as diabetes, heart diseases, and arthritis are effectively self-managed conditions. That is, the individual with the condition makes the day-to-day choices, such as what to eat, when to eat, what activity to do, and if and when to take medication. These choices affect the course of their condition. Maintaining effective self-management over decades and for some an entire lifetime, requires persistent motivation and support, for someone to sustain these daily lifestyle demands, if they are to maintain optimal health and prevent other long-term complications of their condition. In addition, chronic diseases also require individuals to manage their emotional well-being, as they deal with the emotional burden of life impacted by their condition. This adds to the burden of management, low emotional well-being also associated with more adverse outcomes for these conditions (de Ridder et al., 2008; Cal et al., 2015).

How to provide the motivation, information, skills, and support people need, to effectively manage this condition over their lifetime continues to be a vexing problem for health care systems and professionals. The first wave of research on developing these programs for people with chronic conditions focused on the key questions as to whether the programs are effective in improving the health and well-being of attendees. This literature indicates that state of the art programs for chronic disease self-management education and support show some benefits for physical and mental health, at least in the short term (Warsi et al., 2004; Cheung et al., 2011; Nolte and Osborne, 2013). With evidence suggesting these programs can be effective, a second wave of research has moved onto to focus on the delivery of programs to identify what are the active ingredients that distinguish between effective and less effective programs, how they work (Craig et al., 2008) and *how* the interventions are most effectively delivered (Michie and Abraham, 2008).

This is challenging as these self-management and health behavior change programs are inherently very complex (Medical Research Council, 2000). Interventions to change behavior are defined as ‘co-ordinated activities that are meant to change specific behavior patterns’ (Michie et al., 2011). In describing these programs developers and researchers trying to synthesizing the literature have traditionally described the contents of intervention studies by their subject matter (e.g., weight loss, dietary information, parent skills), modes of delivery (e.g., face-to-face, discussion groups, counseling sessions, classes), the theory that they used to inform the study and the professional background of the person delivering the components of the intervention (Davidson et al., 2003). Investigators have attempted to include descriptions of the different techniques the program facilitators or educators have used to promote chronic disease care, such as self-monitoring, reinforcement, or feedback, but had not used agreed set of techniques descriptions across studies (An et al., 2008; Michie et al., 2009; French et al., 2014). This made synthesizing research across studies impossible. It is especially problematic, as the complex nature of these programs means they combine different techniques (such as self-monitoring with goal setting) in different orders and at different times. Thus, it is not possible to establish what was supposed to be delivered in the programs. Therefore, the field of self-management, lifestyle and behavior change research required replicable descriptions of interventions, to enable detailed descriptions of programs to be provided. This would also enable the fidelity of program delivery to be determined for these complex face-to-face interventions. These are essential to enable researchers to compare their effectiveness of different programs, and techniques, to identify their most effective components and ultimately to enable their replication in health care.

In this context, researchers stated that it would be helpful if there existed an agreed set of content descriptions of interventions, in terms of concrete techniques they could translate into practical procedures for the delivery of the intervention (Michie et al., 2013; Hagger and Hardcastle, 2014; Dixon and Johnston, 2021). Otherwise, in the end, they have to guess how the intervention content relates to effectiveness of an intervention study. In response to these calls, researchers have endeavored to list and differentiate a range of Behavior Change Techniques (BCTs) used across these types of interventions. BCTs are considered the smallest components of an intervention and are defined as “an observable and replicable component designed to change behavior” (Michie et al., 2015). One result of this work is the hierarchically structured taxonomy of techniques in behavior change interventions developed by Michie et al. (2013). Using a panel of international expertise in the field to develop a consensus, they identified an initial list of 93 behavior change techniques, clustered into 16 domains. Subsequent groups have identified a number of additional techniques that are used in programs from specific theoretical perspectives.

Having established some common lists of techniques that have consistent definitions and descriptions, another challenge has arisen. That is how the communication of behavior change techniques is implemented in the interaction with the individuals attending the programs (Hagger and Hardcastle, 2014; Hardcastle et al., 2017). This is the focus of this paper: how behavior change techniques are communicated and specifically which theoretical and methodological approach that needs to be taken to understand these professional-client interactions.

The first objective of this paper is to demonstrate that this field of research has to date been based on a monological approach to communication. This will be outlined both theoretically and by demonstrating how studies in behavior change apply the monological approach in their analysis of face-to-face interventions. Thereafter, we argue that the monological approach is problematic and fails to address the key issues this research is concerned with. Therefore, we propose that a different approach is needed, and seek to show that a dialogical approach to behavior change research is required to address the key questions concerning the fidelity of intervention delivery and optimizing the effectiveness of chronic disease self-management programs. That is, we propose a dialogical approach is required, as it builds on the understanding that any communicative exchange is based on a kind of reciprocity of perspectives (Linell, 1998, p.42).

### Monologism

Monologism is a term that has been developed from theoretical assumptions and empirical approaches and that deal with human cognition, communication and language (Linell, 2003).

**Figure 1:**
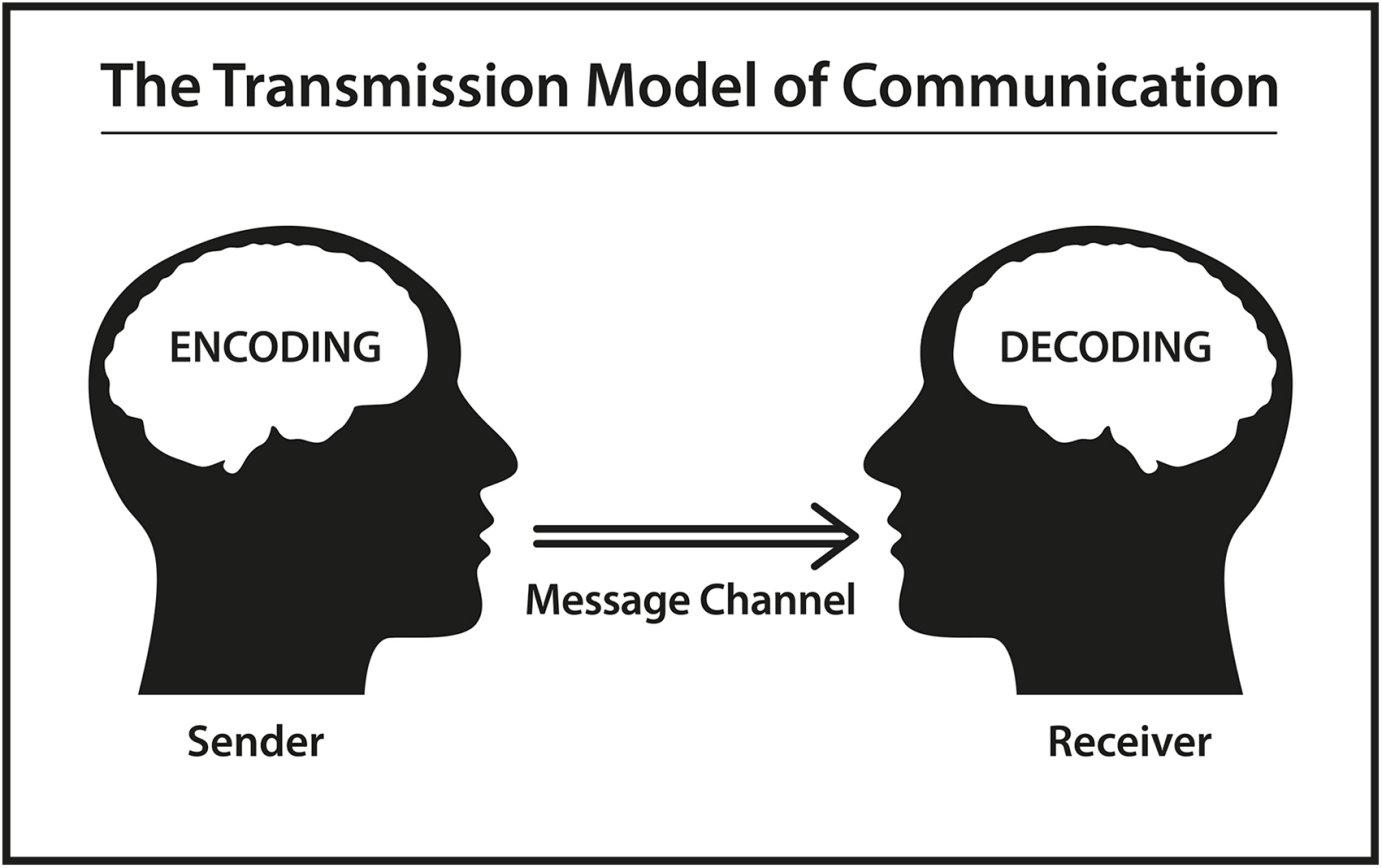
Based on a schematic diagram of a general communication system (Shannon, 2001).

Derived from Greek, Monologism could be translated as “a single voice” (“mónos” Greek “alone,” “lógos” Greek “word, speech, proverb, etc.”) and the paradigm has a history in Western philosophy and theory of the mind (Linell, 2009b; Rommetveit in Wold, 1992). The term seems to have its offspring in philology. The philologist is interested in language and its utterances as entities, who he will examine thoroughly as abstractions (Irving, 2012). The monological approach has its point of departure in the individual, resulting in the presumption that human memory and cognition is more or less a question of storing, recalling and processing collected information. This implies that the human mind holds representations of meanings and that utterances are the product of individual speakers and their minds. Monologism looks at language as a set of basic or derived signs with a predefined meaning and assumes that the individual speaker has to learn and store the important parts of the language system to become a linguistically competent user. As far as communication is concerned, Monologism sees it as a transfer of information from one speaker to another, a so-called “from-to-process,” and interaction is viewed as just sequences of individual actions (Wold, 1992; Linell, 1998). Thus, communication and cognition seem to be distinct processes, since information is seen as transferred, unaffected from one human being to another (Figure 1).

### Behavior change research

The core of behavior change interventions is to ensure a correlation between the intervention techniques used and the desired outcome, with the aim of achieving scientific confidence about effectiveness. Regardless of the core of the behavioral problem the success of behavioral change is measured in quantitative countable entities (e.g., less cigaret smoked, less alcohol consumption, lower saturated fat intake levels, lower BMI or weight loss). The measurable units of an intervention are equated with the behavior to be changed in the intervention.

As health professionals have focused on communicative techniques to convey intervention content and, in extension, they have also turned their attention to how professionals convey it in a relational manner. Therefore, other research groups have described and identified further specifications about the communicative style and techniques that they believe are relevant to interventions. In Hagger and Hardcastle (2014) the providers’ interpersonal style is mentioned as important determinant of intervention effectiveness and refers to the language used in delivering the intervention. The authors exemplify this by emphasizing that controlling language use, which contains auxiliary verbs such as ‘shall’ and ‘must’, should be bypassed.

In continuation, Hardcastle et al. (2017) then report on additional BCTs to those proposed by Michie and colleagues and compendium. Hardcastle and colleagues classify the techniques into categories related to their function as content-based or techniques that are relational techniques. Content-based techniques are specific techniques that are designed to change something, for example action planning to increase someone’s self-efficacy.

In contrast, relational techniques do not serve to target a specific behavior change mechanism, rather they are focused on the developing the relationship by the client and the person delivering the behavior change techniques. Hardcastle and colleagues state that there is a clear difference between relational techniques and the professional’s ‘generic competencies’ (p.5). They emphasize that the professionals’ interpersonal style is the medium used to increase the effectiveness of the content-based techniques. The authors state that an applied relational technique is the professional’s specific action toward the client. Thus, attention is paid to the fact that “the how” of communicating the intervention content could be closely linked to the relationship between the professional and the client.

The study of Hardcastle et al. (2017) attempts to identify overlapping techniques between the complex intervention of Motivational Interviewing (MI) and Michie and colleagues BCTs (2013). The approach of MI was initially described by Miller and Rollnick (2013) and has been applied in different contexts in promoting health behavior change. Hardcastle et al. refer to a meta-analysis of a large number of randomized controlled trials applying MI in “health-related contexts” revealing a higher effectiveness in “improving both behavioral and health-related outcome” compared to usual care. Nevertheless, there also exist studies without any evidence for effectiveness of MI in health behavior changes (Hardcastle et al., 2017, p. 2). Hardcastle et al. sought to identify the specific MI techniques that overlap with BCTs and possibly to identify the techniques that are unique to MI. They note that MI encompasses different BCTs that relate to both the content of a given intervention and to the establishment of a relationship between the professional and the patient. The authors then identify 16 overlapping techniques between BCTs and MI, stating one of the overlapping techniques relational. This study seems to be the starting point that more knowledge of relational techniques could direct the research field of behavior change in the desired direction of more assured replicability.

### Monologism and behavior change research

The monological nature of the existing approach is then highlighted by the growth in meta-analytic and meta-regression papers on these BCTs. Here, authors have systematically identified trials of behavior change interventions, and extracted data on what BCTs were used in the intervention. Most commonly based on descriptions of what was supposed to happen, rather than reports of what actually happened. This results in a list of studies with an accompanying list of BCTs reportedly used in the study. The presence of the BCTs is then entered into meta-analytic statistical models, to identify which BCTs are associated with greater effect sizes in the identified studies. This is done usually in isolation, but some have reported on the combinations of BCTs in relation to their outcome. However, this analysis relies on the theoretical description of interventions found in study reports, and on the presence or absence of studies. There is no attempt to report on the frequency with which a technique is used, the sequences in which techniques are used, or if the technique was actually used consistently across each iteration of the intervention in the studies. Thus, we can see that by looking solely at the delivery of techniques in isolation of their response to the client’s behavior, and independent of the client’s response to a particular technique, this approach is inherently monological.

In extension of Hardcastle and colleagues assumptions on content-based and relational techniques, Dixon and Johnston (2021) aim to develop a framework of competencies in the delivery of behavior change interventions by health professionals. They claim that the competencies of the professionals who convey the intervention content could be decisive for the effectiveness of the intervention. The authors emphasize that some studies have shown better effectiveness of intervention concerning *who*—meaning professional background—delivers the intervention content. They describe a separation between the BCT and the delivery of the technique. Within the delivery component, they also include “communication style.” Dixon and Johnston refer to Dombrowski et al. (2016) who present a Form of Delivery table (FoD) which includes “[…] all the features through which behavior change intervention content is conveyed […].” This table addresses the ‘who’, ‘how’, ‘what’, ‘where’, ‘when’, ‘how much’, ‘tailoring’ and finally ‘style’. In the latter, the authors include the delivery and communication style and communication techniques. They exemplify authoritarian, patient-or asset-centered access and patient or professionally guided communication by applying listening, questioning or reflective communicative techniques.

Thus, it appears from the researchers’ above-mentioned statements and considerations that communication style is constituted by the choice of words and sentences. However, it is unspecified how, e.g., an authoritarian communication style can be identified, and which communication technique primarily shows reflectiveness. If the choice of words and sentences is the starting point for how the respective communication styles are assessed, then there must be taken a closer look at interlocutors’ language in exchange. Several diabetes associations have published guidelines and position statements for how to talk to and about people with diabetes (Speight et al., 2012; Dickinson et al., 2017; Cooper et al., 2018). These guidelines rely on theoretical and philosophical arguments but lack supporting empirical data that specify the use of communicative techniques. Further, these guidelines and the work of researchers focus on delivery, speak only of the language of the health professional. This is therefore inherently a monological perspective that fails to consider the interactional and contextual nature of program delivery.

In the above-mentioned FoD (Dombrowski et al., 2016) the authors use the professionals’ communication intentions as the basis for its evaluation. In the method section by Hardeman et al., this assumption is partially confirmed (2008, p. 14). The authors deliver descriptions of *adherence measures* where they derive a measure for *observed adherence to techniques*. It is not clear which linguistic contributions should be categorized under these techniques in terms of evaluating adherence to techniques.

As mentioned, there is a growing focus on fidelity of delivery in the BC intervention research field. This raises the question of how fidelity of delivery is measured. In a literature review, Walton et al. (2017) searched on measures of fidelity of delivery in BC interventions. The included 66 studies measure fidelity of delivery, engagement or both. Measuring fidelity of delivery is done by using different approaches, varying from researchers coding observational data, quantitatively rated qualitative interviews, client self-reported measures to multiple other measures. In the appendix, Walton et al. summarize how the studies measure the fidelity of delivery of face-to-face health BC interventions. The analyses of most of the studies show that researchers may take the patients’ self-report, the professionals’ coding of the patient by observation or the patients’ contributions collected by interviews into account. Most of the studies cited do not use video or audio recordings as a basis for their analysis.

Interventions in behavioral change are complex (Hawe et al., 2004; Craig et al., 2008). Besides having knowledge concerning the core of interventions (e.g., weight loss, alcohol problems, parental teaching, etc.), it seems more important how the professional conveys this knowledge in a way that is adapted to the individual. It seems surprising that studies of behavior change primarily are interested in whether health professionals use particular enabling techniques, but they seem less attentive to the extent to which participants in an intervention can confirm these techniques in their response. The lack of linking client with professional in the investigation on therapeutic style exposes a monological approach.

The intention of an utterance in Monologism lies in the mind of the speaker and the information appears to be transferred unchallenged between sender and recipient. Having the method of Conversation Analysis (CA) in mind, attention should be payed to the associated responses of turns. We want to demonstrate that a monological approach to the coding of techniques is problematic in terms of fidelity of delivery and ultimately for the desired replicability of interventions.

The following data excerpts come from a medical consultation in diabetes care. They intend to demonstrate that it is problematic to take utterances out of their sequential context when assessing and coding communication techniques. According to the task of the intervention, the health professional (H) asks a client (C) with diabetes to consider starting on insulin in both excerpts.

*Excerpt 1*.

H = health professional; C = client.

1. **H**: May I just check with you, erm why are you not keen on starting on insulin? What.
2. concerns do you have?
3. **C**: Cause I er well I know although I’m a man, I’m like afraid of needles. I mean, yea so, I.
4. do not really dare to poke myself in the tummy to inject the insulin. Yea, that’s one thing.
5. lah, yea.
6. **H**: Mmhmm can I just confirm with you, what are the current medicines you are currently.
7. taking for your diabetes?

*Excerpt 2*.

H = health professional; C = client.

1. **H**: I agree with, its time to perhaps for you to consider to start insulin as an addition to your.
2. treatment to diabetes. How do you feel about that?
3. **C**: I do not want la, always need to inject. Wo pai tong ah, I very scare of pain. As I think of.
4. the needle break inside ah, needle very long ah, cannot cannot.
5. **H**: Mhm mhm, I understand your concerns and it’s not easy um er ah, truly for someone,
6. for anyone actually. To have to use needles to poke themselves on a daily basis. Ermm I.
7. understand that you will… you have a… can I say maybe just feel the fear of the needles la.
8. so… have anyone actually shown you the diabetes… the insulin needle la, that we are using.
9. nowadays?

In excerpt 1 the health professional’s (H) intention in l.1 could be noted as professionally guided communication with a questioning communicative technique to investigate the client’s (C) thoughts about taking insulin. H shows with the question curiosity concerning C’s reluctance to start on insulin. In BCT, H’s action can be registered in accordance to the protocol, because the initial question (l.1–2) would be taken as the basis for evaluation of applied techniques. The same could be said for H’s second utterance (l.6–7) about the patient’s intake of current medicine. H’s action can again be registered in accordance to a protocol that expects the professional to behave in a questioning and patient-oriented manner.

Yet, when ignoring the sequentiality of utterances, the difference of the communicative techniques in H’s first turn (l.1–2) and H’s second turn (l.6–7) will not be captured.

If we take a dialogical perspective, H does not adequately attend to the information in C’s utterance (l.3–5). C’s refusal to inject insulin indicates a fear of needles that seems greater than he can consider the consequences for his health. In the third turn of the sequence (l.6–7), H does not consider C’s uttered fear. Thus, considering the whole sequence (l.1–7), it seems obvious that H ignores C’s emotional concern underlying his utterance (l.3–5). Consequently, in a dialogical perspective, H’s second turn and question is not to be rated as the above-mentioned communicative technique ‘questioning and patient-oriented’.

In excerpt 2, H’s introductory question could again be noted as a professionally directed communication with a questioning technique (l.1–2). As in excerpt 1, H asks about C’s feelings and receives a similar response, since C utters a deep fear of needles (l.3–4). H’s turn (l.5–9) shows how the professionally guided communication with a questioning communicative technique is expanded. H acknowledges C’s feelings by expressing understanding and by pointing out that the feeling is common for many people with diabetes. This is followed by H’s clarifying question that aims at considering C’s fear of needles in order to counteract it. In total H’s utterance—as in excerpt 1—seems to match the original intentions in H’s introductory question but adds the “listening” and “reflecting” to the overall communicative techniques.

Regarding emotional cues and emotional concerns in patient-provider encounters, there has been different definitions of the issues over the decades (Zimmermann et al., 2007). At the third meeting of the Verona network for Sequence Analysis, researchers found consensus on the definition of emotional cues and concerns occurring in medical consultations (Del Piccolo et al., 2005). This resulted in definitions of emotional *cue* as “a verbal or non-verbal hint which suggests an underlying unpleasant emotion that lacks clarity,” and emotional *concern* as “a clear and unambiguous expression of an unpleasant current or recent emotion that is explicitly verbalized with or without a stated issue of importance” (Zimmermann et al., 2011). In addition, this led to a method rooted in the dialogical approach of CA, the Verona Coding Definitions of Emotional Sequences (VR-CoDES), in which the patient’s expressed emotional cues or concerns and the provider’s response to them are qualitatively coded (Zimmermann et al., 2011). With the above definition as a basis, Cs utterances in both excerpts are clearly expressed emotional concerns. There are different reasons why providers’ responses on patients’ emotional cues and concerns are important for the provider-patient interaction and in the end for an intervention (Mjaaland et al., 2011). In behavior change interventions, it is by now common knowledge that clients not only need knowledge of their state of health, they also need a high level of motivation to change their behavior. This can be promoted by specialists and requires them to have communication skills that go beyond the typical conveyance of information and advice (Lawrence et al., 2016). When clients utter emotional concerns or give the professional emotional cues in the course of an intervention, the professional has the opportunity to react on these and thereby create a relational space, which, in turn, opens up the possibility of supporting the client’s motivation for change.

The monological approach on communicative techniques would not capture either the lack or the production of a response on emotional concern in the data excerpts. There are tools available in the literature to code professionals empathic responding to patient cues. However, these are not used in research on the use of BCTs, either content or relational based. If the desired result of the above intervention is, e.g., to convince patients to start taking insulin, then the provider of the intervention counting applied techniques would not know, why some patients could be convinced and some not. Thus, the success of an intervention stays opaque when applying the monological approach on communicative techniques. This is why patients’ utterances and the providers’ reactions must be seen in a sequential perspective, since sequences can reveal changes or expansions in the applied techniques.

These excerpts from a behavior change intervention illustrate that the division into content-based and relational techniques would not necessarily bring us any closer to an understanding of how interventions can be made replicable. Especially not because in a dialogical approach the one technique cannot necessarily be distinguished from the other.

We therefore propose that the research area of health-promoting BC needs to think in dialogical ways in order to be able to approach the goal of replicability. This means that besides having content-related obligations for an intervention, researchers should collect knowledge for the delivery of these contents by paying attention to how the professionals respond to recipients of interventions.

#### Dialogism

Dialogism has its origin in the Russian philosopher Mikhail Baktin’s life work (Bakhtin and Emerson, 1984). Baktin perceived monologization in, e.g., dominant linguistics, philosophy and politics in his contemporary world. Together with his colleague in the 1920s, the linguist Valentin Vološinov, he argued for looking at the applied use of language instead of the predominant theoretical and idealistic understanding of language. With Marxism’s ideology and its philosophical ideas about society and the human being, Bakhtin and Vološinov argued that language should be studied in its applied context—in society and reality—which placed language within an interactional context (Vološinov, 1975). Bakhtin differentiated the theoretical concept of a “sentence,” which by then was treated as an independent analytical object with an independent meaning of content, and the concept of an utterance, which is dependent on the previous utterance. He anchored his argumentation in the dialogical approach on conversation. The contemporanous philosopher Ludwig Wittgenstein supported this dialogical approach by describing language use in everyday life as a “language game” noting that people play by rules in performing verbal actions (Littlejohn, 1992, chap. 5; Wittgenstein et al., 2009). In agreement with Bakhtin and Vološinov, he claimed that the meaning of language depends on the context in which it is used. Starting from Wittgenstein’s philosophical foundation, J.L.Austin and J. Searle developed the Speech Act Theory, stating that utterances are actions of intentional behavior (Searle, 1969; Austin, 2009). Although Speech Act Theory lists various properties of speech and language that contextualize verbal interactions, these mainly concern the individual speaker and portray the listener as a rather passive player (Barron, 2003, chap. 2). Thus, Speech Act Theory does not take the interactive aspect of speech acts into account (Linell and Markovä, 1993).

In Dialogism, cognition is seen as involving intra-and interpersonal communication. Conversely, this implies a cognitive process in any form of communication. While communicating and using our cognition in parallel, we are in dialog with interlocutors and contexts. Inevitably this means that every act involving communication and cognition is seen as something responsive to the context it is embedded in. This leads to the dialogical understanding of ‘conceptual intertwinement’ of cognition and communication. The one notion cannot be explained without mentioning and explaining the other. The monological approach tends to see and explain them as distinct phenomena. The risk arises that one of the terms is assigned a dominance both analytically and conceptually, which, in turn, harbors the risk of analyzing only one of the phenomena.

**Figure 2:**
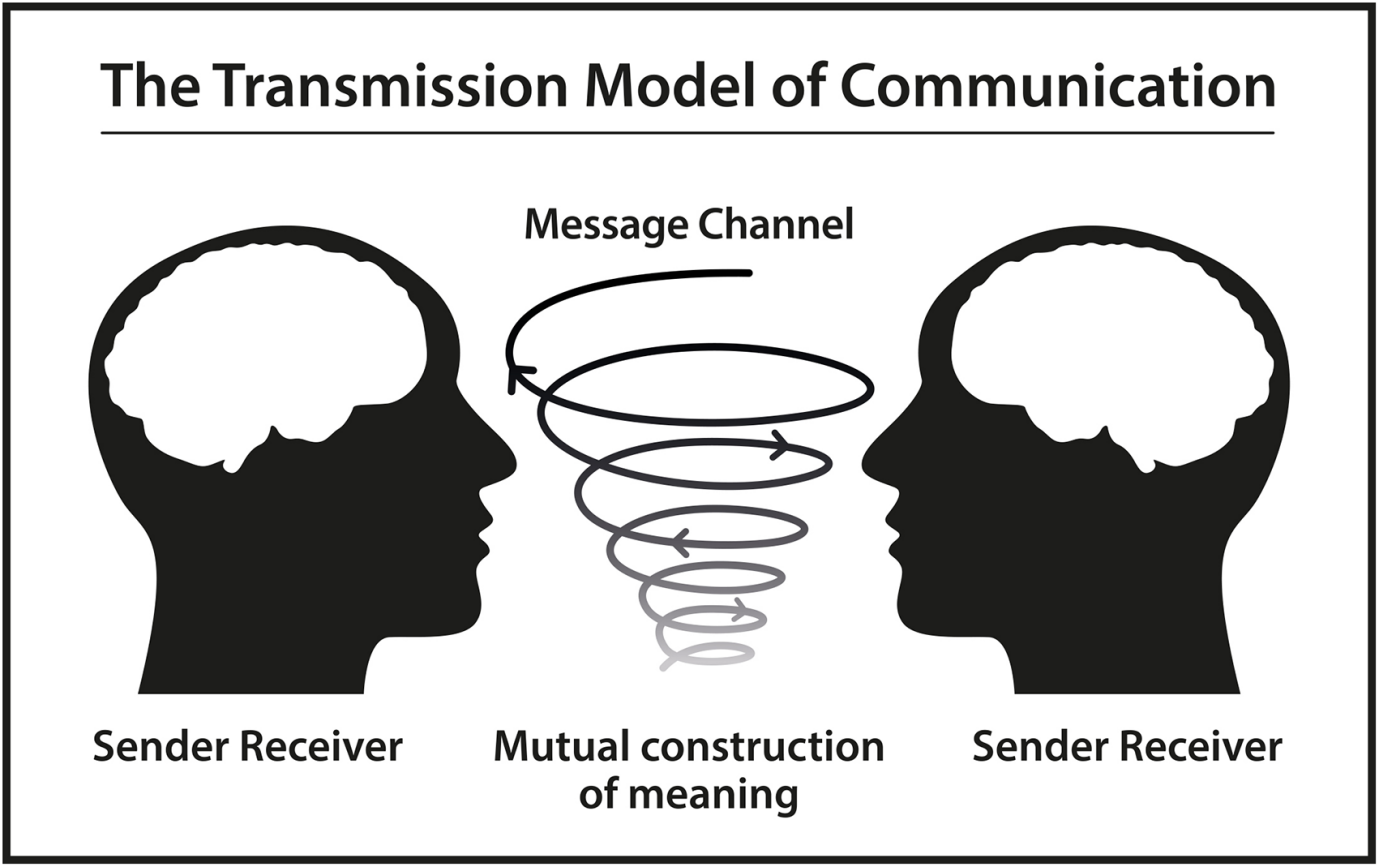
Based on a schematic diagram of a general communication system (Shannon, 2001).

In Dialogism, communication and cognition are seen as being mediated by language. Language is constituted not only by sounds and words or grammar but by utterances that are contextualized and socially and culturally embedded. Hence, the main practice of language is that of conversation and social interaction. Meanings of utterances do not arise separately in the individual and cannot only be assigned to the speaker of an utterance. In the dialogical approach, one assumes that meanings and contents arise through verbalization in conversation with the other. This, in turn, means that the communicative and cognitive act shows the speaker’s perspectivized understanding of something. The listener on his behalf has his own subjective perspectives and tries to understand the speaker’s perspective, who prior to his utterance has tried to attune it to the listener’s perspective. In Dialogism, communication is described as a “between-process” underpinning that communicative acts are dependent on each other (Wold, 1992; Linell, 1998). If one wants to shed light on this ‘between-process’, one should look into research in linguistics and social science (see Figure 2).

#### Content-based and relational techniques—An artificial division

As mentioned, the field of behavior change research has attempted to classify techniques in interventions into content-based and relational techniques. In a dialogical approach, this distinction seems problematic. Regardless of which technique professionals attempt to apply, the meaning of the wording remains in a monological space if not contextualized. If we want to grasp the meaning of linguistic constructions, we should try to understand the relationship between structure, meaning and use of linguistic constructions, which is an endeavor of all grammars. This also applies to construction grammars (CxG), which range from more formalistic to cognitive editions and fundamentally represent the inseparability of form and function of grammatical units. Furthermore, construction grammar theory insists on considering constructions in use (Fried and Östman, 2004). However, linguistic constructions do not necessarily create meaning as a matter of course. Linell emphasizes, even though construction grammars are user-based, they lack the dialogical and thus the contextualized and context-dependent view of linguistic constructions (Linell, 2009a).

The Theory of Functional Linguistics supports this angulation of linguistic meanings. According to functional grammars, they maintain the description of grammatical structures, but state that all grammatical parts of an utterance have semantic, syntactic, and pragmatic functions and that they serve a purpose. With his research, the Australian linguist M.A.K Halliday has made an important contribution to examine and describe the basic functions of language. He gives language two overarching functions: it is meant to give meaning to human experience and to help us carry out our social relationships (Halliday and Matthiessen, 2004, p. 29). Halliday states that the functions are linguistic reflections of how humans use language multifunctional to express meaning. In the first overarching function, the ideational metafunction, language constructs experience. While expressing experience with words in aligned order, we communicate content to each other. However, language also displays our personal and social relationships with our environment. Human language shows how we participate in speech situations and acts. This metafunction is referred to as the interpersonal (Halliday, 1973, p. 41). According to Halliday, our grammar and wording in speech situations will always have a relational meaning beyond the content-related side, because, as he claims: “language is what it is because of what it has to do” (Halliday and Kress, 1976, p. 17). This implies that each time we express ourselves, we create two simultaneous strands of meaning: the ideational (content) and the relational.

We recognize the goal of the research field to strive for a high level of replicability of interventions. The division between content and relation in terms of communication is unlikely to bring us any closer to fulfilling this goal. We rather suggest observing how professionals in interventions act relational. This does not relieve the obligation that providers of interventions should have a basic understanding of what would be beneficial for health behavior change, be it smoking cessation, reduced alcohol consumption, weight loss, etc. We propose that the BCTs can serve as a guide for structuring delivery of a behavior change intervention, but they should not be considered a delivery tool for interventions. In a dialogical approach to language, the effort to pursue and acquire techniques in an intervention and to divide them into content-based and relational ones is missing the point of replicability. If every technique has both a content-based and a relational side within the applied context, then the analytical focus should be on the successful dialog between professional and client in order to gather knowledge on how interventions are successfully delivered.

#### Conversation analysis

Research in social science, language use and social interaction has been grasped by different methodological approaches. One of them is the method of Conversation Analysis (CA), which has its origins in the thoughts of the sociologist and ethnomethodologist Harold Garfinkel. His reflections on social interaction ended with the realization to investigate what people do, rather than gathering reports from interactants about their intended behavior. The sociologists Harvey Sacks, Gail Jefferson and Emanuel Schegloff adopted this approach and found in their studies of recorded interactions that talk is an organized endeavor. Over the decades, CA has been used extensively by researchers and has been developed further in other areas of social studies as Health and Communication, Political Science, Mass Media and Communication, Linguistics, Education and Anthropology (Sidnell and Stivers, 2014, p. 3).

The main interest of CA is the organization of social activity which is considered as collectively organized by the interlocutors of actions. Through many years of study, research in CA has crystallized an overwhelming number of terms that seem to determine the normative structures and interactional rules of social interaction. CA’s very first and pathbreaking observation within social interaction is *sequentiality*. This indicates that utterances have a reflexive relationship both to what is prior and what comes next and that they must be understood in their local context. Strivers (2014, p. 192) mention that many actions in conversation are organized by adjacency pairs, and explain these as actions by which interlocutors require “a normative obligation on co-interactants to perform a type-fitted response at the first possible opportunity” (2014, p. 192). This rule is applied both when producing utterances and when reacting to co-interlocutors’ utterances. A response forms the second part of an utterance, whereas a response that does not occur will be noted as absent -both reactions prompted by the initial utterance. The features of adjacency pairs are mainly as follows: They are composed of two turns, by two different speakers, they are placed adjacent to each other, the utterances are paired to each other and follow one another (pairs e.g.: greeting with greeting, question with answer and others). However, this pairing does not exclude different forms of sequence expansions, which in the end can mess up the adjacent placement of the potential pairs.

Taking sequence expansions into consideration, paired utterances can occur with some time lag and possibly over several intervening utterances. This should be taken into account when assessing adherence to communicative techniques (Hardeman et al., 2008). Consequently, the reaction to an applied technique would therefore not come immediately as a result, but possibly—if at all—with some time delay or over the course of several turns. Therefore, to perform coding requires paying attention to the context in which techniques and the associated responses occur. Using CA as a method, it is possible to study client responses to techniques and, in sequential progression, to study how providers respond to client responses. The answer to the question of *how* to deliver interventions effectively can be addressed here. As a consequence, we should redefine BCTs from being “an observable and replicable component to change behavior” to the point that techniques should be understood as directing “an intended outcome of intervention content through a series of interactions” to capture the dialogical meeting of these encounters.

As behavior change interventions, delivered in person, take place in a social space, they are inherently interactive, evolving, and contextualized, that is dialogical. Yet, the way we assess them, especially for fidelity, is by the above-mentioned monological approach to applied language in interaction.

## Discussion

The road to gaining knowledge enrichment for delivery in interventions can prove rocky and arduous, and the definitive answer on how to gain scientific replicability in detail is beyond the scope of this article. However, we will endeavor to make a suggestion for further work by inquiring what knowledge other domains have acquired by engaging in a dialogical approach.

As previously mentioned, CA’s method is already being used in a wide variety of fields of social studies, including the broad field of psychology. The research of BC in interventions seems far removed from the psychotherapeutic research field. However, if look for parallels, it may turn out that knowledge gained from CA studies in the psychotherapeutic field can help us to better describe and understand processes of change in general.

When Josef Breuer, a colleague of Freud’s, described his therapeutic steps with his patient Anna O, he established an expression that later became the basis of all psychotherapeutic treatment: “talking cure” (Buchholz and Kächele, 2013). Talking and conversing is mainly what happens in therapeutic meetings in order to influence the clients’ cognition concerning their issue at stake. Applying a conversation analytic approach to psychotherapy has opened up the possibility of describing with a higher level of detail how this influence is exerted in the process of therapy sessions. Research in psychotherapy has found the CA method applicable—especially in terms of “sequentiality of turns”—in that “the participants inevitably have to orient to and work with the understandings that each bring about through their actions” (Peräkylä et al., 2008, p. 16). In psychotherapy, the goal is to improve the client’s psychological functioning and health, which ultimately leads to a process of change.

Voutilainen et al. (2018) analyzed tape recorded interactions of three different approaches in psychotherapy with the method of CA in order to investigate how participants demonstrate processes of change. The aim of the study was to use detailed sequence analysis to show how the participants develop over the course of the therapy sessions. The main topics of the respective clients were condensed from the transcriptions and it was documented how the ‘talking about it’ changed in the course of therapy. Methodologically, the authors strived to “show how the microscopic analysis of sequences and the more macroscopic analysis of themes complement each other in research on therapeutic change” (Voutilainen et al., 2018, p. 227).

Peräkylä (2005) performed a conversation analytic study of patients’ responses to interpretations in psychoanalysis therapy. In the psychoanalytic tradition, the therapist’s interpretation of the client’s condition is seen as an aid to expanding *intra*personal insight. In the dialogic understanding of a therapeutic intervention, verbalized interpretations on the part of the therapist are considered an *inter*personal concern at all times. Meaning, that the therapist not necessarily points out discoveries by interpreting the client’s mental state, but tries in alliance with the client to gain and construct a new understanding of the condition. Consequently, from a dialogic perspective in therapy and intervention, there should exist neither the expression “*patient* resistance” nor the need for “*patient* compliance” as both are collaborative constructions of the participants in interactions.

As described in a review on CA studies on psychotherapy, Peräkylä (2019) illustrates with examples from the literature that the methodological approach of CA can help to enlighten the processes of transformation in psychotherapeutic interventions. By creating a simple model of sequential organization of psychotherapy interaction (Peräkylä, 2019, p. 267), the author draws attention to the ‘third position’, explaining that the therapist usually elaborates the client’s response to, e.g., a prior “target action” (e.g., a question). The crux of the ‘third position’ in psychotherapy is that in this part the therapist obviously has the power to influence the transformation of the client’s experiences, be they emotions, relations, or referents. CA’s sequential treatment of contributions is brought to the fore. In addition, an attempt is made to show how psychotherapeutic goals are aimed at with linguistic means and adapted sequences and reactions on behalf of the professional. Based on this research in psychotherapy, it makes sense that something similar should also be investigated in the field of behavioral change research. *How* professionals respond to responses (Peräkylä, 2019, p. 262) may shed more light on how interventions work and what is relationally crucial for them to work. The previously mentioned separation of content-related and relational techniques becomes superfluous, since the “third position” ensures that content and relation merge into one another. As we can learn from the psychotherapeutic field, it seems possible to communicate content relationally and ultimately bring about change.

We know from the therapeutic research field that “little evidence substantiates the benefit of technique-based training” (Ogles et al. 1999, p. 215; Jørgensen and Reppen, 2004, p. 536) and this raises the question to what constitutes the active ingredients in therapeutic interventions that can be linked to changes in the client. Samstag (2002) describes the problematic approach of wanting to locate a specific technique in a specific contact between therapist and client in order to apply this technique in another dyad with other interlocutors involved. According to the author, this will decontextualize what has been effective in parts of a therapy and thereby remove the characteristics “from the fundamental interpersonal context of the therapeutic experience” (p. 59). Jørgensen and Reppen (2004, p. 536) summarizes that the common or non-specific factors in therapy weigh more than technical or specific elements meaning that the therapist’s generic competencies are crucial for a successful process. It emphasizes the importance of the professional being able to adapt relationally to the client. Without a dialogical approach to interaction, this relational alliance, which is decisive for the communication of therapeutic goals and content, cannot be grasped analytically.

The field of behavior change research must consider and decide what to measure, when and by whom in order to classify an intervention as successful. It requires providers to set endpoints for different levels of an intervention (de Haes and Bensing, 2009). When the effectiveness of an intervention is measured against a specific endpoint outcome, e.g., weight loss or glucose levels, this is only one criterion for the success of the intervention. When another endpoint outcome is *how* the intervention is delivered, the measurement of success looks different. Further studies are needed to examine the correlation between different endpoint outcomes and the success of interventions.

## Conclusion

In this paper, we have attempted to make a contribution aimed at learning to look differently at the notion of replicability and at understanding the delivery of interventions in BC research. By exploring the existing literature, we have tried to show that theoretical and methodological frameworks that seem to be anchored in a monological approach to behavior change communication dominate previous research. We have suggested that a dialogical view on the communicative exchange between professional and client would give a greater opportunity to investigate how professionals deliver content in BC interventions. Furthermore, we have argued that BC research views the professionals’ applied BC techniques as functional intentions of their actions. In contrast, we argue that providers of interventions cannot assume that certain professional formulations in disguise of techniques bring about a process of change. Applied techniques do not account for how successful an intervention is delivered. Rather, the approach should be dialogical, since the reciprocity of the dialog provides information about how the interacting parties achieve processes of change together. However, examining these aspects in detail requires not only a different theoretical angle, but also a method that can help to shed light on interactional processes. This is where the application of the CA method becomes relevant, because it opens up the possibility of investigating how participants react linguistically to respective contributions and how these influence each other. This may provide some helpful information about the delivery of the intervention and the provider’s interpersonal style. Further research needs to be conducted to allow for a more accurate description of what appears linguistically and interactionally appropriate in the delivery of interventions.

## Author contributions

AS has outlined, written, and edited the manuscript ready for submission. CF and TS have contributed to the conceptualization and editing. All authors contributed to the article and approved the submitted version.

## Conflict of interest

The authors declare that the research was conducted in the absence of any commercial or financial relationships that could be construed as a potential conflict of interest.

## Publisher’s note

All claims expressed in this article are solely those of the authors and do not necessarily represent those of their affiliated organizations, or those of the publisher, the editors and the reviewers. Any product that may be evaluated in this article, or claim that may be made by its manufacturer, is not guaranteed or endorsed by the publisher.
